# Epidemiological Characterization of Clinical Fungal Isolates from Pauls Stradinš Clinical University Hospital, Latvia: A 4-Year Surveillance Report

**DOI:** 10.3390/life11101002

**Published:** 2021-09-23

**Authors:** Nityanand Jain, Inese Jansone, Tatjana Obidenova, Raimonds Sīmanis, Jānis Meisters, Dagnija Straupmane, Aigars Reinis

**Affiliations:** 1 Department of Biology and Microbiology, Faculty of Medicine, Riga Stradiņš University, Dzirciema Street 16, LV-1007 Riga, Latvia; aigars.reinis@rsu.lv; 2 Joint Laboratory, Pauls Stradiņš Clinical University Hospital, LV-1002 Riga, Latvia; inese.jansone@stradini.lv (I.J.); tatjana.obidenova@stradini.lv (T.O.); janis.meisters@stradini.lv (J.M.); dagnija.straupmane@stradini.lv (D.S.); 3 Department of Infectology, Faculty of Medicine, Riga Stradiņš University, Dzirciema Street 16, LV-1007 Riga, Latvia; raimonds.simanis@stradini.lv

**Keywords:** antifungal resistance, antifungals, opportunistic infections, fungi, *Candida*

## Abstract

Nosocomial fungal infections are an emerging global public health threat that requires urgent attention and proper management. With the limited availability of treatment options, it has become necessary to understand the emerging epidemiological trends, mechanisms, and risk factors. However, very limited surveillance reports are available in the Latvian and broader European context. We therefore conducted a retrospective analysis of laboratory data (2017–2020) from Pauls Stradinš Clinical University Hospital (PSCUH), Riga, Latvia, which is one of the largest public multispecialty hospitals in Latvia. A total of 2278 fungal isolates were analyzed during the study period, with *Candida* spp. comprising 95% of the isolates, followed by *Aspergillus* spp. and *Geotrichum* spp. Amongst the *Candida* spp., *C. albicans* and *C. glabrata* made up about 75% of the isolates. The Department of Lung Diseases and Thoracic Surgery had the highest caseload followed by Intensive Care Department. Majority of the fungal isolates were collected from the bronchoalveolar lavage (37%), followed by urine (19%) and sputum (18%) samples. A total of 34 cases of candidemia were noted during the study period with *C. albicans* being the most common candidemia pathogen. Proper surveillance of emerging epidemiological trends serve as the most reliable and powerful cornerstone towards tackling this emerging threat.

## 1. Introduction

Antifungal resistance (AFR) or antifungal tolerance is an emerging clinical issue globally, which has historically been long been neglected, mainly due to focused attention towards the management and control of resistant bacteria, parasites, and viruses [1,2]. However, with a significant increase in the reported number of cases of systemic fungal infections, the issue of AFR has become a global concern [1,3]. Fungal infections, especially invasive ones, which are associated with a significantly higher mortality rate and a limited availability of treatment options, further worsen the current situation [3]. Additionally, the recent emergence of multi-drug-class-resistant fungal isolates makes the management of such infections even more difficult [3].

In general, fungal resistance can be divided into two broad types: Microbiological and Clinical. Microbiological or in vitro resistance is defined as the nonsusceptibility of a fungal isolate to the tested antifungal drug, whereby the minimum inhibitory concentration (MIC) of the drug exceeds the susceptibility breakpoint for a particular fungal species [1,3]. It can be further divided into intrinsic (or primary) and extrinsic (or acquired/secondary). Clinical or in vivo resistance, on the other hand, is defined as the inability of the antifungal drug to stop the persistence or progression of a fungal infection despite the drug having in vitro activity against the organism [1,4,5].

The development of fungal resistance amongst the isolates depends upon multiple factors, including the fungal factors, host factors, drug-related factors, and environmental factors [1,4,5,6]. Fungal factors include mutations in genetic encoding for efflux pumps [7], target metabolic enzymes [8], biofilm formation [9], modification of plasma membrane composition [8], etc. Amongst the host factors, degree of immunosuppression, site and severity of infection, and timing and dosage of antifungal agents play a critical role in determining the clinical outcome of the treatment [4,6]. Pharmacodynamics and pharmacokinetics, including the fungistatic nature of the drugs, empirical treatment (despite the etiological agent being known), inappropriate dosage, long treatment durations, drug–drug interactions, etc., comprise the drug-related factors that play an important role in the development of fungal resistance [1,7]. Finally, the widespread and indiscriminate use of fungicides in agricultural activities (e.g., the use of azole-based fungicides for cereal and grape cultivation in European member countries) have led to the increased incidence of resistant fungal strains in the environment [1,10].

Nosocomial infections (healthcare-associated infections, or HAIs) broadly refers to the infections that were acquired in a healthcare institution whilst receiving healthcare and which were not present at the time of admission [11]. Since the start of the century, an uptick in the incidence of fungal HAIs has been reported globally, which is most likely the consequence of the widespread use of aggressive treatment modalities [12]. Surgical procedures, such as hematopoietic stem cell transplantation, organ transplantation, chemotherapy, immunomodulatory treatment, and the use of invasive devices, such as intravascular central lines, etc., have been responsible for the increase in fungal HAIs [12,13]. Furthermore, exposure to airborne fungal spores and pathogens within the hospital environment, especially during construction, has further worsened the situation for immunocompromised patients [12,14]. In fact, according to Perlroth et al., the relative frequency of fungal nosocomial infections and the intensity of patient immunosuppression required to predispose a patient to fungal HAI are inversely dependent on one another [15]. For example, candidiasis is mostly seen in patients with relatively minimal immunosuppression, making it the most common nosocomial infection [15,16,17,18], compared with aspergillosis, the second most common nosocomial infection, which tends to occur in patients with moderate to severe immunosuppression [15,16,17,18].

There are three primary classes of antifungal drugs that are used for treating invasive fungal nosocomial infections [19], namely, polyenes, azoles, and echinocandins. Polyenes, such as amphotericin B (AMB) and nystatin (NYS), the latter of which was historically known as fungicidin, were amongst the first broad-spectrum, fungal-specific antibiotics on the market and are the gold standard for the treatment of fungal infections [20,21,22]. AMB, primarily used for systemic mycosis, has a complex mechanism of action that is yet to be fully elucidated. The drug is known to bind with ergosterol (a cholesterol analogue) at the fungal membrane, causing pore formation (leading to rapid depletion of intracellular ions, such as K^+^, Ca^2+^, and Mg^2+^) and sequestration of ergosterol [23,24,25,26]. Additionally, it can induce oxidative damage in the fungal cell [23,27]. Nystatin, due to its poor gastrointestinal absorption, is used only as a topical agent against mucosal infections, such as oral or vulvovaginal candidiasis in an ambulatory setting [28,29]. As with AMB, NYS also induces pore formation, which is accompanied by strong membrane reorganizations, thereby exerting its cytotoxic effects [30].

The azole-class antifungals, since their availability, have become the cornerstone of antifungal treatment and are divided into two subgroups. The older azoles, or imidazoles, such as ketoconazole (KET), econazole (ECO), and miconazole (MCZ), work by inhibiting the synthesis of ergosterol via the inhibition of the enzyme lanosterol demethylase [31,32]. However, as with NYS, due to poor gastrointestinal absorption and adverse side effects during systemic use, their application is limited to the treatment of superficial mycosesand in ambulatory settings [31]. The newer azoles, or triazoles, such as fluconazole (FLU), have a similar mechanism of action whilst having a superior pharmacokinetic and adverse event profile than imidazoles, making them suitable for systemic use [31]. Finally, echinocandins, such as caspofungin and micafungin, are secondary metabolites of fungi that can inhibit the biosynthesis of β-(1,3)-D-glucan, an important cell wall component needed for the maintenance of the structural integrity [33,34]. Apart from these three primary antifungal drug classes, there are two more classes—flucytosine and allylamine—which are used for the management of fungal infections. Flucytosine (FCT) is a pyrimidine analogue that inhibits DNA synthesis. It is converted to 5-fluorouracil which integrates itself during RNA synthesis, leading to early chain termination [34,35]. Allylamines, such as terbinafine, on the other hand, work by inhibiting the enzyme squalene epoxidase, which is involved in the ergosterol synthesis pathway [34,36].

In our recent study, we described the antimicrobial resistance (AMR) rates in Gram-negative bacteria in our hospital from 2017–2020 [37]. Subsequently, in the present study, we aim to discuss and present the epidemiological surveillance data rates during the same period (2017–2020) in our hospital. Due to constant changes in the epidemiology amongst nosocomial infectious agents, it is essential to develop and maintain an updated record of such infectious agents to improve patient care and to develop and put in place proper infection control and prophylaxis strategies.

## 2. Materials and Methods

### 2.1. Study Location and Design

The present study was conducted at the Pauls Stradinš Clinical University Hospital (PSCUH), Riga, Latvia, from 2017 to 2020 (data for full year from January to December was analyzed). A retrospective analysis of the epidemiological data of nosocomial fungal infections, along with the antifungal resistance rates, was completed. The data were collected from the storage server of the Joint Microbiology Laboratory, PSCUH. Microbiological data were collected for patients who fulfilled the ECDC (European Centre for Disease Prevention and Control) criteria for nosocomial infections [38], irrespective of nationality, gender, age, etc. Ethical permission for the present study was granted by the Clinical Research Ethics Committee of the PSCUH, vide no. 290421-16L, dated 29 April 2021.

### 2.2. Sample Collection and Determination of Antifungal Resistance Rates

Clinicians and nurses in their respective departments collected and delivered the patient samples to the Joint Laboratory for microbiological investigations. Specimens collected included abscess material, bronchoalveolar lavage, tracheal aspirate, sputum, blood, urine, etc. Specimen collection was performed based on local and EU guidelines. Specimens with inadequate sample material or improper labelling were removed from the present study. Repeated or different specimens from the same patient which showed the same fungal species were considered as a single isolate in one calendar year (if less than 21 days apart). Specimen handling was performed in accordance with the latest EUCAST (European Committee on Antimicrobial Susceptibility Testing) guidelines [39]. For non-*Candida* spp., species identification was done using a Vitek2 analyzer or MALDI-TOF MS (matrix-assisted laser desorption ionization-time of flight mass spectrometry). For *Candida* spp., the Candifast test (ElitechGroup, Puteaux, France, https://www.elitechgroup.com/product/candifast, accessed on 20 September 2021) was used for species.

### 2.3. Data Collection and Analysis

The laboratory database was perused for data regarding antifungal resistance, sample types, species, and the department from where the samples were collected. The data was then downloaded for the period 2017–2020.

## 3. Results

### 3.1. Candida *spp*. Were the Most Frequently Isolated Nosocomial Fungal Species

During the study period, a total of 2278 (567 in 2017, 533 in 2018, 629 in 2019, and 549 in 2020) distinct fungal samples were collected which belonged to 10 different fungal genera (Table 1). *Candida* spp. was the most isolated nosocomial fungal species, representing more than 95% of the total isolates in the study period. *Aspergillus* spp. (2.59%) and *Geotrichum* spp. (1.28%) were the second and third most isolated species, respectively. Since the isolated samples of *Aspergillus*, *Fusarium*, *Cryptococcus*, etc., were extremely sporadic and isolated (only 104 isolates over 4 years, which was on average two cases per month for all species other than *Candida*) and no identifiable clustered outbreak in the hospital was noticed, an empirical standard treatment regimen based on local and EU guidelines was followed for these patients. Hence, we concentrated only on the *Candida* spp. in the results section, whilst a brief overview of other fungal agents is provided in the discussion section.

### 3.2. Characterization of Candida *spp*. Isolates

Further, as shown in Table 2, amongst the *Candida* spp., *C. albicans* represented 68.37% of the isolates, followed by *C. glabrata* (8.46%) and *C. tropicalis* (8.14%), respectively. In the case of *Aspergillus* spp., *A. fumigatus* was more frequently isolated than *A. niger*.

### 3.3. Distribution of Candida Isolates Based on Department and Patient Specimen Collected

**Figure 1:**
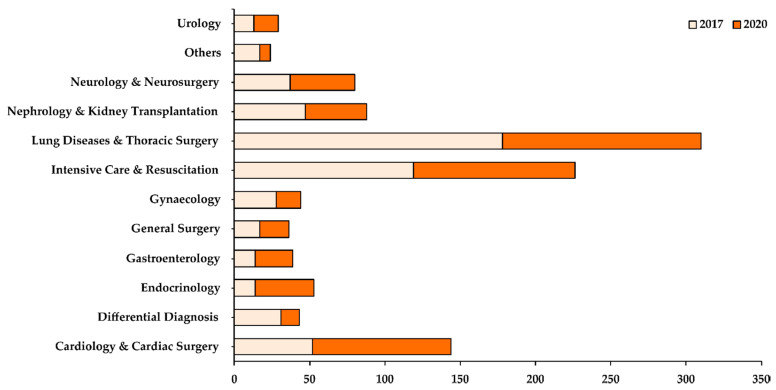
Distribution of fungal samples isolated based on different departments in 2017 (light orange) and 2020 (dark orange). The X-axis shows the total number of samples collected while the Y-axis shows the different departments in the hospital.

The highest number of nosocomial fungal isolates were cumulatively collected from the Department of Lung Diseases and Thoracic Surgery (Figure 1). Although the department saw a decrease in the number of cases in 2020 when compared with 2017, it still accounted for 28% of all samples collected in 2017 and 2020. The Intensive Care Department was the department with the second-highest caseload of nosocomial fungal infections (20%).

**Figure 2:**
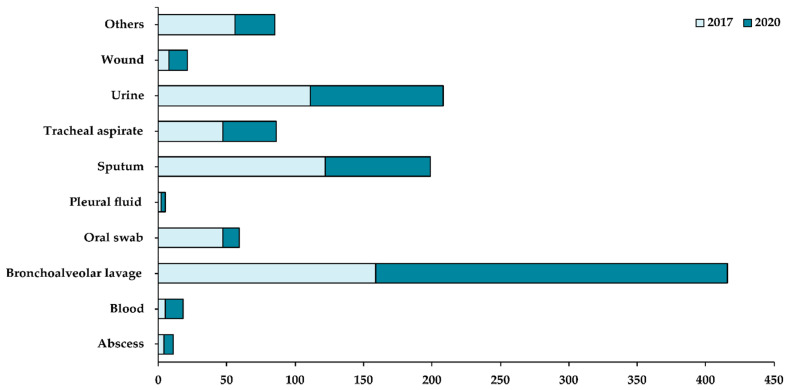
Distribution of fungal samples isolated based on patient specimens collected in 2017 (light blue) and 2020 (dark blue). X-axis shows the total number of samples collected while the Y-axis shows the different patient specimens collected in the hospital.

Whilst most of the departments reported a decrease in their caseload in 2020 when compared with 2017, some departments, such as Urology, Cardiology & Cardiac Surgery, Endocrinology, and Gastroenterology, showed an increase in their caseload in 2020 (Figure 1). Based on the patient specimens collected (Figure 2), most of the fungal isolates were collected from the bronchoalveolar lavage (37%), followed by urine (19%) and sputum (18%) samples. A small number of fungal isolates were collected from the blood, abscess, and pleural fluid of patients.

### 3.4. Distribution of Candida Isolates Based on Cases of Candidemia

A total of 34 cases were reported for fungal blood infections (Table 3) in the study period. Amongst the *Candida* spp., in terms of the causative agents of invasive fungal infections (blood infections), *C. albicans* was the most common causative fungal pathogen, followed by *C. glabrata* and *C. tropicalis*. Apart from candidemia, one case of *C. neoformans* and two cases of *S. cerevisiae* in 2019 were also reported as causative agents for invasive fungal infections.

## 4. Discussion

Nosocomial fungal infections are an emerging global threat that leads to additional hospitalization costs, longer treatment durations, and are associated with higher mortality rates [40]. According to recent estimates in 2017, fungal infections create an economic burden of more than USD 7.2 billion (United States Dollars) on the healthcare system in the USA alone [41]. Further, in an educational survey conducted by the CDC (Centers for Disease Control and Prevention), more than two-thirds of the respondents failed to recognize any of the six common fungal infections listed in the survey [42]. This prompted the CDC to launch the “Think Fungus” yearly campaign to educate and spread awareness regarding various fungal infections [43]. Fungal diseases usually present with symptoms that are similar to those of other infections, which makes it difficult to establish the proper diagnosis, leading to delayed treatment, poor patient outcomes, and unnecessary medical costs [15,41]. Furthermore, nonidentification of etiological agents leads to the prescription of empirical treatments and/or the initiation of prophylactic treatments for high-risk group patients [15]. Finally, antifungal drugs usually target both human and fungal cells (both cells are eukaryotic) which leads to multiple adverse side effects in patients, and limits the available targets for the development of newer drugs [44,45,46]. All these reasons make the proper management of microbiological AFR the need of the hour.

Since comprehensive international or national databases are usually not available for most of the fungal infections, analyses of databases of large hospitals and laboratories can enable us to predict and estimate the national and local caseload besides examining the shifting trends in AFR amongst fungal isolates. Hence, studies akin to the present one provide an informative overview of the present situation and highlight the need for the establishment of comprehensive surveillance protocols and treatment guidelines. Furthermore, the present study can be utilized for spreading awareness amongst the hospital staff and management regarding various fungal infections and etiological agents.

### 4.1. Candida *spp*. as Nosocomial Fungal Agents

Amongst the over 200 recognized *Candida* spp., many species can cause candidiasis, a broad term referring to infections ranging from superficial cutaneous and mucosal infections to deep-seated organ infections [47]. Candidiasis can occur at any age and usually occurs in conditions with identifiable infection risk factors [47]. As part of the normal gastrointestinal and genitourinary microflora, many members of the genus *Candida* are known to cause opportunistic infections, especially in the setting of immunosuppression [44,48]. The members are known to be the causative agents of vaginitis, oral candidiasis, cutaneous candidiasis, candidemia (bloodstream infection), and other systemic infections [49]. *C. albicans* is the most identified nosocomial fungal agent, followed by *C. glabrata*, *C. tropicalis*, *C. parapsilosis*, and *C. krusei*. Together it is estimated that these five members are responsible for up to 90% of invasive infections, although their distribution and caseload vary based on geographical region, patient population, and clinical settings [50,51,52].

In our present study, as shown in Table 2, we found that whilst *C. albicans* was the most isolated *Candida* spp. (68%), the burden of *C. glabrata* and *C. tropicalis* was rather similar (about 8% each). Apart from these five members, which are also found in normal healthy individuals, the emergence of other species, such as *C. kefyr* and *C. dubliniensis*, has also been reported worldwide [51,53]. In fact, in our hospital, they together comprised about 4% of the total *Candida* isolates and showed an increasing trend of incidence in 2020 when compared with 2017. There are multiple risk factors for the spread of nosocomial candidiasis, including overuse of broad-spectrum antibiotics, immunosuppression, chronic malignancy, surgical intervention, parenteral feeding, burns, premature neonate, diabetes, and/or prolonged hospitalization [54]. Their ability to colonize and survive in various habitats makes it easy to spread the infection, especially within the hospital environment, where *Candida*’s members have been reported to survive for up to 4 months [55]. Additionally, another group of researchers reported that infection can also spread via the hand-to-hand contact route due to their ability to survive for about 45 min on peoples’ hands after inoculation [56].

The primary event for candidiasis is the colonization of the host by the yeast cells. The cells usually adhere to the host cells and produce hydrolytic enzymes [57]. Adherence prevents (or at least slows down) cell clearance, while the hydrolytic enzymes facilitate adherence, tissue penetration, invasion, and subsequent delivery of toxins into the host cell [45,58]. Biofilm production additionally provides protection from the host’s defense response besides conferring antifungal resistance [59]. Apart from the above virulence factors, it is difficult to identify specific *Candida* spp. diagnostically. For example, the specialized culture media and many commercially available analysis equipment do not readily differentiate many *Candida* spp. [60,61,62], thereby delaying the initiation of precise antifungal treatment, a leading cause of poor patient outcomes with nosocomial fungal infections.

### 4.2. Aspergillus *spp*. as Nosocomial Fungal Agents

As with the genus *Candida*, genus *Aspergillus* comprises over 185 members, about 20 of which are implicated in human disease and infection [63]. *A. fumigatus* is mostly isolated in patients with invasive infection, while *A. flavus* is mostly associated with sinusitis [63]. Other emerging nosocomial *Aspergillus* species include *A. terreus* and *A. niger* [63,64,65], in line with the findings from our hospital (*A. fumigatus* was more isolated than *A. niger*). Though primarily found in decaying vegetation, outdoor soil, bird droppings, and hay barns, *A. fumigatus* can also be found in human habitations. Dust particles, infrequently cleaned places, potted flowers, shutters, hard to reach and clean places, such as attics, ventilation ducts, ceilings, etc., are common places of habitation [63]. Some studies have also demonstrated colonization of foodstuff, such as peppers, biscuits, fruits, tobacco, marijuana, etc., by *A. fumigatus* [66,67]. Additionally, construction, renovation, or demolition work in the ward or near the hospital campus can passively disseminate fungal conidia and then be transported by wind and convection currents [68,69].

Airborne transmission and subsequent inhalation of fungal conidia is the main route of infection [70,71]. Due to its small size, the fungal spores colonize the upper and lower respiratory tract, especially the pulmonary alveolar spaces, which serves as the optimum environment for the spores to germinate and form hyphae, ultimately leading to pulmonary aspergillosis [69,72]. Apart from the inhalation route, reports of direct contamination of the wounds or skin in burn patients or low-birth-weight babies have also been published in the literature [66,73]. Whilst transmission by contaminated water and/or aerosolization of spores remains a subject of debate [74], direct contact with contaminated adhesive tapes, gauze, and intravascular catheters provides other routes of nosocomial transmission [66,75].

### 4.3. Geotrichum *spp*. as Nosocomial Fungal Agents

*Geotrichum capitatum* (*Blastoschizomyces capitatus*), previously known as *Trichosporon capitatum*, is an opportunistic invasive fungal infection-causing nosocomial fungal agent. The fungus is part of the normal digestive, respiratory, and cutaneous microflora, which act as ports of entry for the fungus to cause opportunistic infections [76,77]. The genus, although reported rarely globally, is relatively more common in Europe, especially in and around the Mediterranean region (suspected geographical domination in the region is due to climatic factors that favor fungal growth) [78]. However, cases have been also reported from the USA and southern India [78,79]. As with other fungal infectious agents, immunosuppression is the key risk factor, with some authors linking contaminated milk and polytrauma as potential risk factors for infection [80,81,82,83]. Geotrichosis clinically presents similarly to invasive candidiasis, except that the focal point of infection is the lungs. Pulmonary infections and pneumonia are the hallmarks of geotrichosis, which are relatively uncommon in invasive candidiasis [84]. Diagnosis and identification of geotrichosis are standardly based on a positive GM (galactomannan) assay test and pulmonary lesions if fungal isolates are recovered from respiratory cultures [83]. There is usually cross-reactivity with the aspergillus GM assay test, making a diagnosis of invasive geotrichosis even harder [85].

### 4.4. Other Nosocomial Fungal Agents

Amongst other nosocomial fungal agents reported in the present study, *Cryptococcus* spp., represents one of the traditional fungal agents, including *Candida* spp. and *Aspergillus* spp. *C. neoformans* is an encapsulated fungus that usually spreads using an airborne route, with the infection being usually asymptomatic [86,87]. Naturally, the fungus is found in the soil throughout the world and clinically manifests (as pneumonia and meningitis) typically when the latent infection gets reactivated due to immunosuppression [86,88]. In our hospital, all cases were reported from the Department of Lung Diseases and Thoracic Surgery. *Curvularia* spp. is a genus of filamentous pigmented molds that generally colonize the soil and vegetation [89]. The mold can cause infections in both immunosuppressed and non-immunosuppressed patients. In the latter group of patients, *Curvularia* spp. is known to cause infections of the paranasal sinus, skin, nails, nail beds, and soft tissue [90,91,92]. Nosocomial infections, however, can range from invasive and allergic sinusitis, bronchopulmonary disease, ocular infections, peritonitis, and postsurgical endocarditis [89,93].

*Fusarium* spp. is an opportunistic pathogen that causes a wide variety of diseases in humans. In normal healthy individuals, it has been implicated in causing superficial infections, such as skin infections, keratitis, and onychomycosis [94,95,96]. In patients with severe immunosuppression, *Fusarium* spp. can cause invasive infections (both local and disseminated) [96]. Among its virulence factors, the production of mycotoxins stands out. This enables the fungus to suppress humoral and cellular immunity, whilst also causing local tissue breakdown [97]. In the hospital environment, *Fusarium* spp. has been isolated in and around the water distribution system, including water storage tanks, showerheads, sink drains and faucets, etc. [98]. *Pichia* spp. represent another genus of opportunistic pathogens that are normally found to be part of the normal microbiota of the skin, the throat, and the gastrointestinal tract [99]. As a rarely described clinical nosocomial agent, *Pichia* spp. are usually associated with invasive infections and fungaemia in neonates and immunosuppressed individuals [99,100,101]. Standard fungal risk factors, such as prematurity, low birth weight, long duration of hospital stay, prior use of antibiotics, intravenous catheterization, intravenous drug abuse, etc., are associated with Pichia infection [101].

*Rhodotorula mucilaginosa* is another opportunistic nosocomial fungal species, with most infections associated with central venous catheters in immunosuppressed hematological patients [102,103]. Some estimates put the incidence rate of *Rhodotorula* fungaemia at around 0.5% to 2.3% in western countries [104], in line with the findings of the present study. Apart from fungaemia, less invasive localized infections include endophthalmitis, onychomycosis, meningitis, prosthetic joint infections, and peritonitis in both immunocompromised and non-immunocompromised patients [103]. In the environment, *R. mucilaginosa* is commonly isolated from various food items and beverages, including peanuts, apple cider, cherries, fresh fruits, fruit juice, cheese, sausages, etc. [103].

*Saccharomyces cerevisiae*, commonly known as Baker’s yeast or Brewer’s yeast, is usually considered a safe, nonpathogenic organism, with widespread use in the baking, fermenting, and wine industries. However, with the recent advent in molecular detection techniques and increase in the number of immunocompromised patients, the incidence of *S. cerevisiae* as a nosocomial fungal agent has only increased [105]. The infections caused can range from vaginitis and cutaneous infections in healthy individuals to systemic invasive infections in compromised patients (elderly, premature babies, HIV positive patients, etc.) [106,107]. Unlike its phylogenetically close relative, *Candida* spp., *S. cerevisiae* shows low adherence to the host tissue and can only cause infections if the integrity of the epithelial/mucosal barrier is compromised [108]. Finally, *Trichoderma* spp., which are rapidly growing molds, are extremely rare yet emerging nosocomial fungal agents. The infection usually presents with nodular pulmonary infiltrations, peritonitis, cutaneous lesions, and CNS infections, primarily in immunocompromised patients [109].

### 4.5. Antifungal Treatment Principles

The first and most critical step towards proper management of nosocomial fungal infections is the determination of whether the antifungal agents being prescribed are meant for treating mucosal or systemic infections [48]. Whilst superficial infections can be effectively managed using topical preparations, systemic infections require either oral or intravenous (IV) preparations. This is especially important since some antifungal agents are available only for IV infusions (e.g., echinocandins, amphotericin B), or only as oral agents (e.g., flucytosine) [48]. Some drugs can be administered using both routes, however, and therefore the choice would be dictated by considerations of drug solubility (e.g., azoles) [48,110]. The next and most crucial step is to understand the route of clearance of the antifungal agent. For example, fluconazole is excreted in its active form in urine, making it an appropriate drug for the treatment of urinary tract fungal infections [48].

Broadly, amphotericin B remains the gold standard for treatment for most of the mycoses, although its association with nephrotoxicity and the possibility of only systemic administration limits its potential [23]. However, newer formulations of AMB, such as liposomal AMB and lipid complex AMB, have shown promising results with lower toxicity. However, their use is restricted by their high cost [23]. The next in line is the azoles, especially KET and MCZ, which provide a viable and effective alternative to AMB [111]. The triazoles are, however, now preferred over imidazoles due to their low cost, superior efficacy, and better toleration [112]. However, azoles themselves are associated with hepatotoxicity and are victims of increasing antifungal resistance [44]. The newer generation of azoles, such as voriconazole and posaconazole, have been developed and approved for clinical use. These are broad-spectrum antifungal agents which inhibit fungal cytochrome P450-mediated 14-alpha lanosterol demethylation, causing structural damage and a loss of cell membrane function [113]. Both are recommended as first-line prophylaxis against invasive *Candida* and *Aspergillus* infections, while the are second-in-line for treating fusariosis (in the case of intolerance to amphotericin B) [113].

Another treatment strategy includes using antifungal combination therapy, especially with flucytosine, due to its role in hepatic impairment, interference with bone marrow function, and the rapid occurrence of resistance amongst isolates [114,115]. Combination with FCT reduces its toxic effects and is generally clinically used with AMB and FLU. Combination with non-antifungal drugs, such as calcineurin inhibitors, proton pump inhibitors, immunomodulators, etc., have shown promising results. Cyclosporin A, for example, increases the susceptibility of fungal infections to fluconazole by deletion of efflux pumps or alteration of cellular stress responses [116]. The development of newer drug classes, including echinocandins, allylamines, etc., provides a viable alternative to these traditional antifungal agents. The development of ibrexafungerp (formerly known as SCY-078) has shown promising results for the treatment of echinocandin-resistant fungal isolates, especially *C. glabrata*-caused vulvovaginal candidiasis [117]. It is the first oral (1-3)-β-D-glucan synthase inhibitor (GSI) which works by hindering fungal cell wall synthesis [118].

### 4.6. Antifungal Resistance Mechanisms

Antifungal drug resistance (AFR) in fungal isolates is based on different mechanisms depending on the antifungal drug being overused (only a short overview is provided here). Resistance to azoles is governed by three different mechanisms [48]. The first mechanism is the reduction of the concentrations of the drug that is accumulated intracellularly. This is achieved by a gain-of-function mutation in transcription factors that control efflux pump activity (*TAC1* and *MRR1* in *C. albicans*; *PDR1* in *C. glabrata*; *Cdr1p* homologue in *C. neoformans*; *AfuMdr1p* and *AfuMdr2p* in *Aspergillus* spp.) [119,120,121,122]. The second mechanism is decreased affinity for the drug target, for example, mutations in the *ERG11* gene (encoding for lanosterol 14-*α*-demethylase) which increase resistance to azoles [48,123]. The third and final mechanism is counteracting the effects of the drug, which might include an increase in target protein concentrations and/or alterations in the protein structure [123,124].

Although resistance to polyenes (AMB; NYS) is minimum in comparison to azoles, it is achieved by using the reverse mechanism to that used against azoles. An acquired loss-of-function mutation in *ERG3* or *ERG6* genes, both involved in the biosynthesis pathways of ergosterol, leads to a decrease in concentrations of the target protein, thereby providing resistance [125,126]. Resistance to flucytosine is akin to that of polyene and is based on the inactivation of different enzymes in the pyrimidine pathway [48]. Point mutations in *FUR1* and *FCY1* genes lead to disruption of the pyrimidine pathway, thereby conferring resistance [126]. An alternative mechanism to resistance to FCT is via upregulation of efflux pumps due to mutations in the *FCY2* gene [126].

### 4.7. Antifungal Stewardship, Infection Control, and Future Strategies

Planning, development, and implementation of a nationwide (or at least at the level of the hospital), comprehensive antifungal stewardship program (AFSP) is required to tackle the emerging threat posed by the increase in nosocomial fungal infections, along with increasing resistance and the emergence of new infectious agents. An AFSP based on the recently released core recommendations for antifungal stewardship by the Mycoses Study Group Education and Research Consortium [127] will be planned and implemented in the hospital. The plan will include the core “essential” recommendations from the consortium, including [127]:

(i) The development of institutional treatment guidelines for prophylaxis and empiric therapy, including the identification of high-risk patients, the estimation of a proper dosage, the timely identification of the agent, etc.;
(ii) The development of targeted education programs for appropriate diagnosis and treatment for clinicians, specialists, nurses, etc.;
(iii) An antifungal prescription review for drug–drug interactions, including the identification of over-prescribed agents and the rationalization of prescription strategies;
(iv) The development, encouragement, and adoption of an intravenous-to-oral antifungal drug transition program;
(v) Local surveillance and reporting of invasive fungal diseases to prescribers, management, and other relevant health monitoring bodies at the national and EU/EEA level to contribute towards a comprehensive national database.

Our laboratory and hospital provide reference and educational/training services to multiple regional microbiological laboratories and clinics. The findings of the present study will be shared with such interested laboratories, along with the provision of an option to participate in the AFSP program. Within our hospital, the findings will be shared with laboratory specialists, infectious disease specialists, hospital managers, etc., during our regular meetings to formulate further plans. Apart from pharmacological control, nonpharmacological hygienic techniques will be strictly enforced, which can help us go a long way in the prevention of infection spread and decrease antifungal resistance rates.

Proper handwashing techniques (i.e., the six-step handwash), trimmed nails, regular bathing, and full drying of the skin, use of nonocclusive shoes, absorbent socks, powder, and avoidance of sharing of toiletries, beddings, etc., [128] will be strictly enforced and encouraged to prevent the inter- and intra-departmental spread of infection. Since the present study represents the first of its kind in Latvia, it will aid in the establishment of baseline caseload and resistance rates for future studies and surveillance reports.

### 4.8. Limitations of the Present Study

The results obtained in the present study are limited by several of the following limitations. Firstly, we did not perform antifungal resistance analysis. Although Candifast test was used for identification of resistance, the susceptibility profile and clinical practice application of this commercial kit is not well established. Furthermore, multiple studies have advised avoiding the kit for antifungal resistance profiling [129,130]. Secondly, data analysis on fungal isolates from outpatient departments was not performed (to identify community epidemiology rates). Thirdly, being a single-center study, the results may not be completely reflective of the on-ground national situation. Finally, the present study used Candifast test for susceptibility detection, use of which remains controversial in the literature and hence, we suggest the readers to interpret the results of the present study regarding AFR rates with appropriate caution. However, our study marks a beginning and provides other healthcare institutions and laboratories with the means to investigate and report their respective data, which could aid in the curation of a national database for fungal infections. Monitoring of such a curated database could prove to be instrumental in improving patient care by fast-tracking diagnosis and the implementation of proper infection control measures.

## 5. Conclusions

The curation of comprehensive national and international surveillance databases concerning nosocomial fungal infections is the cornerstone to the emerging threat of nosocomial infections. 

*Candida* spp. remained the most isolated nosocomial fungal species, followed by *Aspergillus* spp. and *Geotrichum* spp. *C. albicans* was the most common species amongst *Candida* spp., while a comparable burden of *C. tropicalis* and *C. glabrata* was noted in our hospital.

## Figures and Tables

**Table 1:** Prevalence of different nosocomial fungal genera from 2017–2020.

Genus	Prevalence (%)				Overall Prevalence (%)
	2017	2018	2019	2020	
*Aspergillus* spp.	00.00	03.19	03.34	03.83	02.59
*Candida* spp.	99.82	96.62	94.43	91.08	95.44
*Cryptococcus* spp.	00.00	00.00	00.16	00.18	00.09
*Curvularia* spp.	00.00	00.00	00.16	00.00	00.05
*Fusarium* spp.	00.18	00.00	00.00	00.00	00.05
*Geotrichum* spp.	00.00	00.19	00.95	04.00	01.28
*Pichia* spp.	00.00	00.00	00.16	00.00	00.05
*Rhodotorula* spp.	00.00	00.00	00.16	00.00	00.05
*Saccharomyces* spp.	00.00	00.00	00.48	00.91	00.35
*Trichoderma* spp.	00.00	00.00	00.16	00.00	00.05
**Total (%)**	100.00	100.00	100.00	100.00	100.00

**Table 2:** Prevalence of different *Candida* species from 2017–2020.

Species	Prevalence amongst *Candida* isolates (%)				Overall Prevalence (%)
	2017	2018	2019	2020	
*C. albicans*	74.20	69.92	65.82	63.14	68.37
*C. dubliniensis*	00.00	01.36	03.03	02.00	01.61
*C. glabrata*	08.48	06.99	08.42	10.00	08.46
*C. inconspicua*	00.00	00.00	00.34	01.60	00.46
*C. kefyr*	00.71	01.94	02.36	06.21	02.71
*C. krusei*	03.36	03.88	05.05	04.01	04.09
*C. lusitaniae*	00.18	00.19	01.01	00.80	00.55
*C. parapsilosis*	01.24	02.52	03.03	03.01	02.44
*C. tropicalis*	08.30	09.71	08.42	06.02	08.14
Others	03.53	03.49	02.52	03.21	03.17
**Total (%)**	100.00	100.00	100.00	100.00	100.00

**Table 3:** Number of cases of candidemia based on species from 2017–2020.

Species	2017	2018	2019	2020	Overall
*C. albicans*	3	4	2	9	18
*C. glabrata*	1	3	3	0	7
*C. parapsilosis*	0	1	0	0	1
*C. tropicalis*	0	1	2	1	4
*C. krusei*	0	0	1	0	1
*C. inconspicua*	0	0	0	2	2
*C. lusitaniae*	0	0	1	0	1
**Total cases**	4	9	9	12	34

## Data Availability

All data analyzed in the present study are presented in a summarized manner in the results section of the manuscript. Patient data cannot be provided due to confidentiality and privacy concerns.
